# Venous thromboembolism risk factors and usefulness of a risk scoring system in lower limb orthopedic surgery: A case-control study in Japan

**DOI:** 10.1097/MD.0000000000028622

**Published:** 2022-01-28

**Authors:** Akihiko Akamine, Naonobu Takahira, Masayuki Kuroiwa, Atsushi Tomizawa, Koichirou Atsuda

**Affiliations:** a Orthopedic Surgery, Clinical Medicine, Graduate School of Medical Sciences, Kitasato University, Sagamihara, Kanagawa, Japan; b Department of Pharmacy, Kitasato University Hospital, Sagamihara, Kanagawa, Japan; c Physical Therapy Course, Department of Rehabilitation, Kitasato University School of Allied Health Sciences, Sagamihara, Kanagawa, Japan; d Department of Anesthesiology, Kitasato University School of Medicine, Sagamihara, Kanagawa, Japan; e Research and Education Center for Clinical Pharmacy, Division of Clinical Pharmacy, Laboratory of Pharmacy Practice and Science 1, Kitasato University School of Pharmacy, Tokyo, Japan.

**Keywords:** prophylaxis, pulmonary embolism, risk assessment, risk factor, venous thromboembolism

## Abstract

We previously developed a computerized clinical decision support system based on national consensus guidelines and previous studies. This system was used to assess the risk of venous thromboembolism. In this study, we examined the risk factors for venous thromboembolism in patients who underwent lower limb orthopedic surgery using our risk scoring system, to investigate the association between the total risk score and the occurrence of venous thromboembolism.

We retrospectively evaluated the records of 649 patients who underwent lower limb orthopedic surgery at a tertiary care center in Japan between January 2015 and August 2018. Venous thromboembolism was confirmed using ultrasonography or computed tomography angiography. The computerized clinical decision support system was used throughout the hospitalization period. Independent risk factors for postoperative venous thromboembolism were identified using logistic regression analysis.

Age (≥68 years) was significantly associated with an increased risk of venous thromboembolism (adjusted odds ratio: 1.06, 95% confidence interval: 1.03–1.09; *P* < 0.001). Furthermore, the Cochran–Armitage trend test revealed a significant positive correlation between the total risk score and the occurrence of venous thromboembolism (*P* < 0.001).

Our risk scoring system may be used preoperatively to determine the need for venous thromboembolism prophylaxis. This study suggests that age (≥68 years) may be a risk factor for venous thromboembolism after lower limb orthopedic surgery. Additional studies are needed to validate these results.

## 1 Introduction

Venous thromboembolism (VTE), including deep vein thrombosis (DVT) and pulmonary thromboembolism (PTE), is a common complication among patients undergoing total knee arthroplasty (TKA) or total hip arthroplasty (THA). VTE affects millions of people globally and is a leading cause of long-term morbidity and short-term mortality.^[1]^ Further, the management of VTE is associated with increased total hospital costs.^[2]^

A recent study^[3]^ showed that VTE occurs in 10% to 40% of patients under medicine and general surgery who do not receive appropriate thrombotic prophylaxis. Additionally, despite substantial evidence of a prophylaxis-associated reduction in the occurrence of VTE, large prospective studies^[4–11]^ show that it is underutilized for patients at risk, with only 20% of eligible patients receiving prophylaxis. Correctly identifying patients at risk of VTE is critical to reducing its occurrence. However, determining the level of VTE risk is complex because of the large number of known risk factors, with each factor conferring a different relative risk but having a cumulative effect when combined with other factors.^[12,13]^ Accordingly, some individualized VTE risk assessment models have been developed clinically, the most famous being those by Cohen et al,^[5]^ Caprini and Hyers,^[6]^ Kucher et al,^[14]^ and Rogers et al ^[15]^ These risk assessment models classify patients into 1 of 3 or 4 risk levels and determine the onset, intensity, type, and duration of prophylaxis.^[3]^

In 2008, we developed a point-scoring, system-based, computerized clinical decision support system (CCDSS)^[16]^ for assessing VTE risk factors, and for selecting the appropriate prophylaxis for VTE. The relative scores for individual risk factors were added to produce a total risk score (TRS) that indicated the patients VTE risk level. The CCDSS provides appropriate recommendations for VTE prophylaxis according to the VTE risk level. However, a few patients who underwent lower limb orthopedic surgery and for whom the CCDSS was applied still developed VTE. Many CCDSS interventions that were previously reported also had limitations as they used the authors own institutional electronic health records, targeted populations, or single-center design.^[17–19]^ We previously reported the efficacy and safety of a portable intermittent pneumatic compression device for patients who are at a high risk of VTE and bleeding.^[20]^ However, the association between the CCDSS TRS and the occurrence of VTE has yet to be investigated.

The primary objective of this study was to examine the risk factors for VTE among patients who underwent lower limb orthopedic surgery in whom the CCDSS was applied. The secondary objective was to investigate the association between the CCDSS TRS and the occurrence of VTE.

## 2 Methods

### 2.1 Study design and patients

This case-control study (level of evidence: III) study was approved by the Institutional Review Board for Observation and Epidemiological Study of the University of Kitasato (approval number: KMEO B20-325) and was conducted in accordance with the guidelines for Good Clinical Practice and the principles of the Declaration of Helsinki. Due to the retrospective nature of the study, the Institutional Review Board waived the need to obtain informed consent from the participants.

We notified or disclosed information about the conduct of the study, and patients were provided with ample opportunities to opt-out. All data used in the study were anonymized. The subjects were patients who underwent lower limb orthopedic surgery at Kitasato University Hospital in Kanagawa Prefecture, Japan, between January 1, 2015 and August 31, 2018. The hospital is a 1185-bed tertiary medical care center and a comprehensive teaching hospital. The inclusion criteria were as follows:

(1) use of the CCDSS before surgery;
(2) age ≥16 years;
(3) hospitalization duration ≥4 days, and
(4) history of lower limb orthopedic surgery (eg, THA, TKA, hip fracture surgery, and other surgery).

The exclusion criteria were as follows:

(1) underwent surgery with VTE;
(2) received prescribed warfarin or other anticoagulants during pharmacologic prophylaxis;
(3) underwent multiple surgeries during the hospitalization period;
(4) had severe renal dysfunction (creatinine clearance <30 mL/min);
(5) had no record of preoperative and postoperative ultrasonography;
(6) CCDSS applied only postoperatively, and
(7) switched from enoxaparin or edoxaban as pharmacologic prophylaxis to another anticoagulant after surgery.

### 2.2 Anticoagulant prophylaxis protocol

Patients were identified as receiving pharmacologic prophylaxis if they received any anticoagulant at a prophylactic dose during their hospitalization period. We administered 20 mg of enoxaparin subcutaneously (Clexane; Sanofi-Aventis, Paris, France) once or twice daily or 15 or 30 mg of edoxaban orally (tablet) (Lixiana; Daiichi-Sankyo, Tokyo, Japan) once daily. Pharmacological prophylaxis was administered for a maximum of 14 days.

### 2.3 Data collection

Data on age, sex, TRS, body mass index (BMI), CCDSS VTE risk level, diabetes, heart failure, hypertension, hyperlipidemia, type of surgery, and development of DVT and PTE after lower limb orthopedic surgery were collected from electronic medical records between February 2018 and July 2019. Two researchers (MO and AA) independently reviewed the data. We defined the duration of observation as the hospitalization period for each patient.

### 2.4 Definition of VTE

DVT was defined as a new thrombus within the venous system after surgery and was recorded after confirmation by vascular ultrasonography (Aplio^TM^ Platinum Series; Canon, Tochigi, Japan). Vascular ultrasonography was conducted by a clinical laboratory technician within 7 days of surgery, and the results were evaluated by a physician.

PTE was defined as a thrombus in the pulmonary artery with subsequent obstruction of the blood supply to the lung parenchyma. PTE was recorded after confirmation using computed tomography angiography (SOMATOM Definition Flash; Siemens Healthineers, Forchheim, Germany). The patients were assessed for PTE at the discretion of the physician when they presented with early symptoms of PTE or had confirmed DVT. Computed tomography angiography was performed by a clinical radiologist, and the results were evaluated by a physician.

VTE was defined as the occurrence of either symptomatic or asymptomatic DVT or PTE, previously defined as a postoperative complication. The patients were divided accordingly into the VTE and non-VTE groups.

### 2.5 Development of the VTE risk scoring system

The CCDSS was developed as a convenient tool for clinicians, using national consensus guidelines and previous studies.^[21,22]^ The CCDSS was shown to clinicians via an electronic medical chart and was available in all hospital wards. The CCDSS was used throughout the hospitalization period. After surgery, the patients risk of developing VTE was evaluated weekly.

The CCDSS is a decision-making system that works in 3 steps.^[20]^ First, the VTE risk level in each type of surgery is categorized. Second, the system automatically adds the risk score for 16 additional VTE risk factors (eg, background factors and patient history) to determine the VTE risk level, and the risk factors are listed with weights of −2 to 8 points each. Finally, the VTE risk level (none, low, moderate, high, or highest risk) and recommended VTE prophylactic methods are shown in electronic medical records. Data on VTE risk factors were collected from the electronic medical records. Patients were also classified into 4 groups according to the TRS as follows: group 1, TRS of −2–1; group 2, TRS of 2–3; group 3, TRS of 4–5; and group 4, TRS ≥6.

### 2.6 Sample size calculation

We used standard methods to calculate the sample size needed for multiple logistic regression, with at least 10 outcomes needed for each independent variable included in the analysis.^[23]^ With an expected VTE incidence rate of 9% to 15%, we required 400 to 670 patients (60 VTE) to conduct multiple logistic regression appropriately with 6 variables. Because our orthopedic department specializes in the treatment of hip joints rather than knee joints, we assumed that the number of THA patients would be 3 to 4 times the number of TKA patients.

### 2.7 Statistical analyses

The risk factors for VTE and the association between the CCDSS TRS and the occurrence of VTE were analyzed statistically. Multivariate logistic regression models were used to examine the effect of independent variables on the development of VTE. The a priori variables were selected based on previous literature^[21,24]^ and included high risk comorbidities and type of surgery associated with the development of VTE. We used Fisher exact test and chi-square test (without the Yates correction) to compare the categorical variables between the VTE and non-VTE groups. Meanwhile, the Mann–Whitney *U* test was used to analyze continuous variables. For the evaluation of TRS characteristics, we defined the TRS as a continuous variable. Normally distributed quantitative data were expressed as means, whereas non-normally distributed quantitative data were expressed as medians. Categorical data were expressed as absolute values and percentages.^[25]^ Significant values (*P*-value = .15) in the univariate analysis were entered into the final multivariate model.^[26]^ Patients with missing relevant data were excluded from the multivariate analysis. Although it did not meet all of the criteria already described, we included the TRS in the final multivariate logistic regression model. We used variance inflation factors to examine multicollinearity. The predictive and complex characteristics of the models were also considered during modeling.

To evaluate the cutoff for those who developed VTE based on continuous variables with significant differences in the multivariate analysis, the receiver operating characteristic curve and area under the curve were calculated.^[27]^ The cutoff values were determined using receiver operating curve analysis. The sensitivity, specificity, positive predictive value, and negative predictive value were calculated for the occurrence of VTE. The distribution of the occurrence rate of VTE in the TRS groups and the significance of differences were reported using Fisher exact test. If significant differences among the groups were identified, post-hoc tests were performed using Holm method to determine which group differed from the other groups. We assessed trends in the occurrence of VTE in the TRS groups using the Cochran–Armitage trend test.^[28]^

All statistical analyses were performed using EZR 13-6 (Saitama Medical Center, Jichi Medical University, Saitama, Japan) and JMP 13.2.1 (SAS Institute Inc., Cary, NC). EZR is a graphical user interface for R (The R Foundation for Statistical Computing, Vienna, Austria). More precisely, it is a modified version of R commander that is designed to add statistical functions that are frequently used in biostatistics.^[29]^ All tests were 2-tailed. A *P*-value of <.05 was considered statistically significant. However, all *P*-values other than those for primary endpoints are nominal *P*-values because the sample size was not calculated.

## 3 Results

### 3.1 Patient characteristics

**Figure 1:**
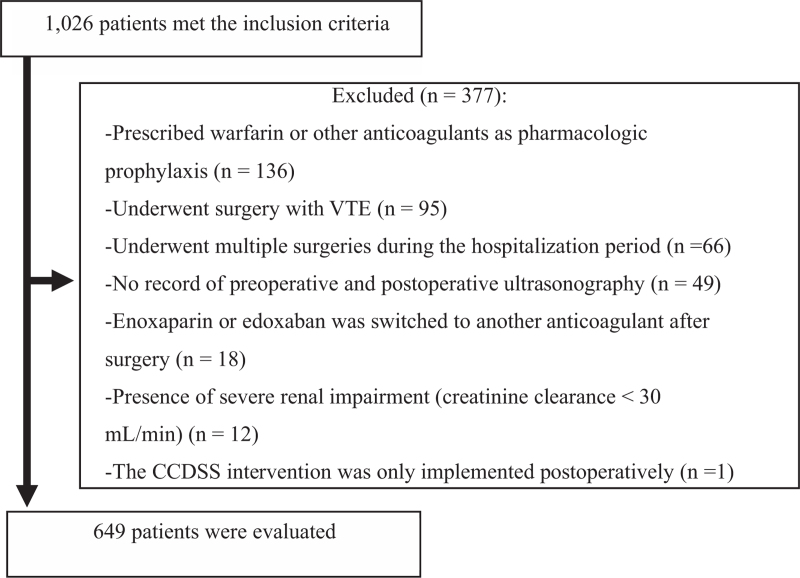
Patient inclusion flowchart. CCDSS = computerized clinical decision support system, VTE = venous thromboembolism.

After applying both inclusion and exclusion criteria using the CCDSS, a total of 649 patients were enrolled in this study (Fig. 1).

Table S1, Supplemental Digital Content shows the baseline patient characteristics. In total, 74 (11%) and 575 (89%) patients developed VTE and did not develop VTE during hospitalization, respectively. The median age of the overall population was 66 (range, 57–74) years, and the VTE group was significantly older than the non-VTE group (median age: 74 vs 65 years; *P* < .001). There was a significant difference in the sex distribution (*P* = .082), TRS (*P* = .002), number of patients with diabetes (*P* = .045), number of patients with hypertension (*P* = .046), number of patients with a history of VTE (*P* = .069), and type of surgery (*P* < .001) between the 2 groups. Meanwhile, the BMI, rate of heart failure, and rate of hyperlipidemia were not significantly different. A total of 480 patients underwent THA, 140 patients underwent TKA, and 29 patients underwent other surgeries. PTE was identified in 1 patient who underwent TKA.

Thirty-six patients each in the THA and TKA groups experienced VTE, while 444 and 104 patients in the THA and TKA groups, respectively, did not experience VTE.

The incidence rate of VTE was higher in patients who underwent TKA than in those who underwent THA (36/140 [26%] vs 36/480 [7.5%]; chi-square test, *P* < .001).

### 3.2 Risk factors and CCDSS scores

Table S2, Supplemental Digital Content shows the number (percentage) of patients who developed VTE according to each risk factor in the CCDSS model. The most common risk factors were age ≥60 years (66/74 [89%]) and BMI ≥25 to <30 kg/m^2^ (23/74 [31%]) Among the 74 patients with VTE, 5 (6.8%) had a history of VTE.

### 3.3 Multivariate logistic regression analysis of risk factors for postoperative VTE

In total, 6 factors were significant on univariate analysis: sex, age, TRS, diabetes, hypertension, and a history of VTE. Multivariate logistic regression analysis showed that age was an independent risk factor for VTE (*P* < .001) in patients who underwent lower limb orthopedic surgery (Table 1). The variance inflation factor showed that there was no collinearity in the model, with none of the variance inflation factor values reaching 10.

**Table 1:** Multivariate logistic regression analysis of risk factors for postoperative VTE in patients who underwent lower limb orthopedic surgery and were managed with CCDSS.

Risk factor	*P*-value	Odds ratio (95% CI)	VIF
Sex	.11	1.82 (0.87–3.83)	1.00
Age (yr)	**<.001** ^∗^	**1.06 (1.03–1.09)**	1.11
TRS	.40	1.08 (0.90–1.29)	3.17
Diabetes	.39	1.27 (0.73–2.20)	1.09
Hypertension	.50	1.22 (0.68–2.20)	1.08
History of VTE	.85	0.84 (0.13–5.34)	3.02

CCDSS = computerized clinical decision support system, CI = confidence interval, TRS = total risk score, VIF = variance inflation factor, VTE = venous thromboembolis.

∗ Significant (*P* < .05).

### 3.4 Age cutoff for the risk of VTE

**Figure 2:**
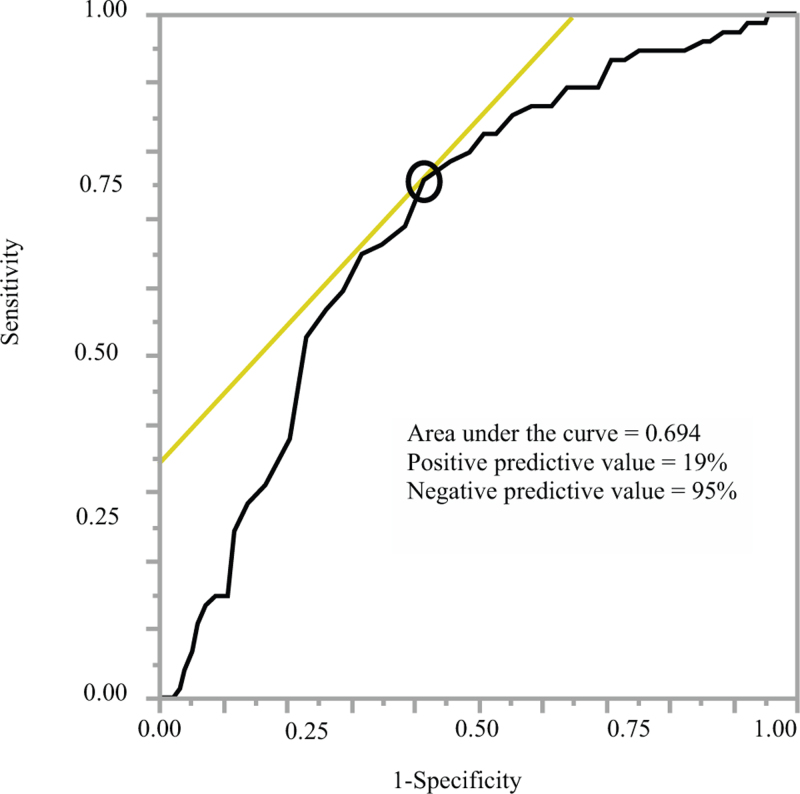
Receiver operating characteristic curve for age as a risk factor for postoperative VTE. The black circle indicates the cutoff point (68 yr). VTE = venous thromboembolism.

The receiver operating characteristic curve showed that the optimal age cutoff for the occurrence of VTE (Fig. 2) was 68 years (area under the curve: 0.694, 95% confidence interval: 0.636–0.752), above which patients were at an increased risk of VTE. The area under the receiver operating characteristic curve was < 0.7, which indicated that the predictive power of the model was low (sensitivity: 76%, specificity: 58%, positive predictive value: 19%, and negative predictive value: 95%).

### 3.5 Correlation between the TRS and the occurrence of postoperative VTE

**Figure 3:**
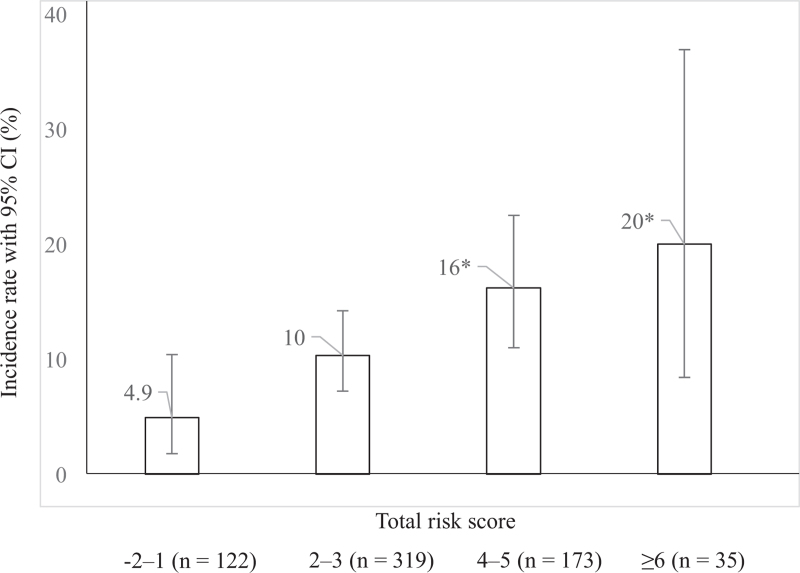
Correlation between the TRS and the occurrence of VTE. The error bars show the 95% CIs. Fisher exact test shows a *P*-value of .005. Holm method was used to correct for multiple comparisons. The Cochran–Armitage trend test shows a *P*-value of <.001; ^∗^*P* < .05 vs group 1. CI = confidence interval, TRS = total risk score, VTE = venous thromboembolism.

Figure 3 shows the correlation between the TRS and the occurrence of postoperative VTE. The incidence was lowest in group 1 (4.9%), whereas it was highest in group 4 (20%). The incidence of VTE was significantly higher in groups 3 (16%; *P* = .017) and 4 (20%; *P* = .048) than in group 1. However, there was no significant difference in the incidence of VTE between groups 3 and 4 (*P* = .62). However, the Cochran–Armitage trend test showed that the incidence of VTE in each group increased with increasing TRS (*P* < .001).

## 4 Discussion

This study examined the risk factors for VTE and the association between the TRS of our risk scoring system and the occurrence of VTE among patients who underwent lower limb orthopedic surgery. We found that the occurrence of VTE in Asian patients was associated with the TRS. To our knowledge, this is the first study that demonstrates such a relationship. Multivariate logistic regression analysis showed that age increased the risk of VTE, and the occurrence of VTE increased with increasing TRS. Age, but not the TRS, was an independent risk factor affecting lower limb orthopedic surgery.

Age is a risk factor for VTE^[21]^ Consistent results were obtained in our study, with 68 years being the optimal cutoff. A previous meta-analysis involving 1,031,683 patients who underwent THA or TKA^[24]^ showed that age was an independent risk factor for VTE. A retrospective study of 505 patients who underwent THA in Japan^[30]^ also showed the same results. Collectively, these findings indicate that VTE prophylaxis should always be considered in older patients, particularly those aged ≥68 years.

The Caprini risk assessment model for VTE is a widely used and clinically validated tool for assessing >40 risk factors in the surgical setting in Western populations.^[31]^ However, as the number of risk factors increases, the assessment of VTE risk becomes more complicated. Moreover, the reliability of the Caprini risk assessment model among Asian patients who underwent lower limb orthopedic surgery has not been fully evaluated. The CCDSS is based only on 16 risk factors. This low number of VTE risk factors in the CCDSS enables physicians to conduct easier risk assessments. Furthermore, it can be used in all patients and may be helpful in determining appropriate preventive measures without compromising safety.

A previous retrospective database study by Bahl et al^[3]^ established the validity of a VTE risk scoring system that was developed based on the Caprini risk assessment model. Further, the results supported the importance of estimating the individual patient risk for VTE in patients who underwent general, vascular, or urologic surgery. However, data on the benefits of a VTE risk scoring system in patients who underwent lower limb orthopedic surgery are limited. Our findings are consistent with a previous study^[32]^ that reported the occurrence of an increased VTE risk with increasing risk score. The incidence of VTE was only 4.9% in group 1 (ie, those with a cumulative risk score of −2–1), whereas a previous study^[32]^ reported a 20% incidence of VTE in their lowest risk score group (ie, those with a cumulative risk score of 0–1). The difference may be attributed to the risk assessment system used and the study patients investigated. The risk assessment system of the previous study used 8 risk factors, with patients only suspected of having VTE. Each patient may have had risk factors that were not identified by either system. In this study, the incidence of VTE was significantly higher in groups 3 and 4 than in group 1. This shows that more attention should be paid to VTE prophylaxis in patients with a high TRS, particularly those with a TRS of ≥4. However, patient distribution according to the TRS was unequal in this study, with the number of patients decreasing as the TRS increased. Further studies with an equal number of patients in each TRS group are needed to better clarify the relationship between the TRS and the occurrence of VTE.

DVT occurs in 42% to 57% of THA patients and in 41% to 85% of TKA patients in the absence of antithrombotic prophylaxis.^[21]^ In this study, 36 of 480 THA patients (7.5%) and 36 of 140 TKA patients (26%) developed VTE. In a randomized controlled trial of 832 patients in Japan,^[33]^ conventional venography in proven VTE occurred in 18 of 90 THA patients (20%) and 25 of 84 TKA patients (30%) despite anticoagulation treatment with subcutaneous enoxaparin (20 mg) twice daily. A systematic review of randomized controlled trials^[34]^ demonstrated that DVT occurred in 338 of 1455 THA patients (23%) and 1046 of 2912 TKA patients (36%) who were prescribed warfarin as a pharmacologic prophylaxis. These VTE incidence rates are higher than those in this study. The difference may be attributed to the use of conventional venography for screening, which detects small thrombi with high sensitivity. However, the CCDSS may be helpful for understanding the VTE risk level of individual patients and for providing appropriate preventive measures according to the VTE risk level. A critically important finding is that older Asian patients, particularly those aged ≥68 years, or with a TRS of ≥4, are strongly considered for adequate VTE prophylaxis after lower limb orthopedic surgery. In the future, we aim to use artificial intelligence to verify the physical data of patients undergoing lower limb orthopedic surgery and the occurrence of VTE, and to create a global VTE risk assessment model.

Preventing VTE has many benefits, including reduced hospitalizations and healthcare costs. An observational cohort study^[2]^ estimated that the attributive cost of a VTE complication is USD $18,310. Therefore, VTE should be prevented during the early stages of hospitalization in accordance with the individualized VTE risk level. In this context, the CCDSS may be deemed useful.

This study has some limitations. First, this was a single-center study, and institution-specific factors may limit generalizability. A similar multicenter study should be performed to establish the association between the risk of VTE and the risk scoring system. A more extensive study has the advantage of reducing confounding factors and increasing generalizability. Second, because of the lack of randomization, our study had some confounding factors. Our sample size is comparable with most previous studies, and the study population was adjusted for a large number of confounding factors. However, we could not adjust for all confounders; thus, our results are not as robust as those from a randomized controlled trial. Third, according to the inclusion criteria, only patients who underwent lower limb orthopedic surgery were enrolled in this study. Moreover, the exclusion criteria limited the number of patients with risk factors for VTE. Hence, the patient selection criteria limited the correct evaluation of the occurrence of VTE risk factors. Fourth, our study included only Japanese participants. A previous study showed that Americans aged >65 years were more likely to be either overweight (BMI ≥25 to <30 kg/m^2^) or obese (BMI ≥30 kg/m^2^) than Japanese individuals of the same age.^[35]^ Furthermore, Asians have a lower risk of VTE than Caucasians.^[36]^ Therefore, ethnic differences should be considered when using the CCDSS and, more importantly, when interpreting our findings. It is still unclear whether using the CCDSS will help reduce the incidence of VTE and promote the use of appropriate thromboprophylaxis. Future studies should evaluate the efficiency of the CCDSS in all patients.

In conclusion, our findings indicate that patients aged ≥68 years who underwent lower limb orthopedic surgery may have an increased risk of VTE. Moreover, the incidence of VTE and the TRS were directly correlated. Accordingly, the incidence of VTE was significantly higher in patients of groups 3 (TRS 4–5) and 4 (TRS ≥6) than in patients of group 1 (TRS -2–1). The CCDSS may be an effective tool for evaluating the risk of VTE in patients who will undergo lower limb orthopedic surgery and may assist physicians in providing appropriate thromboprophylaxis to vulnerable patients.

## Acknowledgments

The authors wish to thank the Working Group of the Deep Vein Thrombosis Prevention Committee at Kitasato University Hospital for their clinical assistance with this research. The authors also thank Masumi Ozaki for her assistance with data collection. The authors would like to thank the surgeons who performed orthopedic surgery at Kitasato University Hospital. The authors would also like to thank Editage (www.editage.jp) for English language editing.

## Author contributions

**Conceptualization:** Akihiko Akamine, Naonobu Takahira.

**Data curation:** Akihiko Akamine, Naonobu Takahira.

**Formal analysis:** Akihiko Akamine, Naonobu Takahira.

**Investigation:** Akihiko Akamine.

**Methodology:** Akihiko Akamine.

**Project administration:** Akihiko Akamine, Naonobu Takahira.

**Supervision:** Naonobu Takahira.

**Visualization:** Akihiko Akamine.

**Writing – original draft:** Akihiko Akamine.

**Writing – review & editing:** Naonobu Takahira, Masayuki Kuroiwa, Atsushi Tomizawa, Koichirou Atsuda.

## Supplementary Material

Supplemental Digital Content

## Supplementary Material

Supplemental Digital Content
