# Supplementary material for: Venous thromboembolism risk factors and usefulness of a risk scoring system in lower limb orthopedic surgery: A case-control study in Japan
Source: Medicine (Baltimore). 2022 Jan 28;101(4):e28622. doi: 10.1097/MD.0000000000028622 (PMC8797501; doi:10.1097/MD.0000000000028622)
Supplement: Supplemental Digital Content [file medi-101-e28622-s001.docx]

**Supplementary Material**

**Supplemental Digital Content (tableS1).** Baseline patient characteristics

| Characteristic | Total  (n = 649) | VTE group^a^  (n = 74) | Non-VTE group  (n = 575) | p-value |
| --- | --- | --- | --- | --- |
| Sex (male)^b^ | 128 (20) | 9 (12) | 119 (21) | 0.082^*^ |
| Age (years)^c^ | 66 (57–74) | 74 (68–77) | 65 (56–73) | <0.001^*^ |
| BMI (kg/m^2^)^c^ | 23.9 (21.4–27.0) | 24.4 (22.3–26.85) | 23.8 (21.3–27.05) | 0.162 |
| TRS^d^ | 2.64 ± 2.25 | 3.46 ± 2.65 | 2.53 ± 2.17 | 0.002^*^ |
| Diabetes^b^ | 166 (26) | 26 (35) | 140 (24) | 0.045^*^ |
| Heart failure^b^ | 14 (2.2) | 1 (1.4) | 13 (2.3) | 0.61 |
| Hyperlipidemia^b^ | 68 (10) | 8 (11) | 60 (10) | 0.92 |
| Hypertension^b^ | 128 (20) | 21 (28) | 107 (19) | 0.046^*^ |
| History of VTE^b^ | 21 (3.2) | 5 (6.8) | 16 (2.8) | 0.069^*^ |
| Type of surgery^b^ |  |  |  |  |
| THA | 480 (74) | 36 (48.6) | 444 (77.2) | <0.001^*^ |
| TKA | 140 (22) | 36 (48.6) | 104 (18.1) |  |
| HFS | 13 (2.0) | 1 (1.4) | 12 (2.1) |  |
| Other^e^ | 16 (2.5) | 1 (1.4) | 15 (2.6) |  |

^*^Significant (p < 0.15). P-values for age, BMI, and TRS were calculated using the Mann–Whitney U test; those for sex, diabetes, heart failure, hyperlipidemia, hypertension, history of VTE, and type of surgery were calculated using Fisher’s exact test

^a^DVT (n = 73); DVT + PTE (n = 1)

^b^number (percentage)

^c^median (interquartile range)

^d^mean ± standard deviation

^e^Rotational acetabular osteotomy (n = 7), joint extraction (n = 3), arthroscopic surgery (n = 2), total hip arthroplasty for hip fracture (n = 2), and open surgery for fracture and debridement

Abbreviations: BMI, body mass index; DVT, deep venous thrombosis; HFS, hip fracture surgery; PTE, pulmonary thromboembolism; THA, total hip arthroplasty; TKA, total knee arthroplasty; TRS, total risk score; VTE, venous thromboembolism
