# Supplementary material for: Venous thromboembolism risk factors and usefulness of a risk scoring system in lower limb orthopedic surgery: A case-control study in Japan
Source: Medicine (Baltimore). 2022 Jan 28;101(4):e28622. doi: 10.1097/MD.0000000000028622 (PMC8797501; doi:10.1097/MD.0000000000028622)
Supplement: Supplemental Digital Content [file medi-101-e28622-s002.docx]

**Supplementary Material**

**Supplemental Digital Content (tableS2).** Proportion of VTE patients (n = 74) by risk factor and CCDSS score

| Risk factor | Point for each risk factor | n | % |
| --- | --- | --- | --- |
| Age <40 years | -2 | 0 | 0.0 |
| Cancer chemotherapy | +1 | 0 | 0.0 |
| Estrogen treatment | +1 | 0 | 0.0 |
| Lower limb motor paralysis | +1 | 0 | 0.0 |
| Oral contraceptives | +1 | 0 | 0.0 |
| Severe infection | +1 | 0 | 0.0 |
| Varicose veins | +1 | 2 | 2.7 |
| Age ≥60 years | +2 | 66 | 89 |
| BMI ≥25 to <30 kg/m^2^ | +2 | 23 | 31 |
| Cancer^a^ | +2 | 0 | 0.0 |
| Lower limb cast bandage fixation | +2 | 0 | 0.0 |
| Confined to bed <48 hours | +2 | 3 | 4.1 |
| Pelvic mass disease | +2 | 3 | 4.1 |
| BMI ≥30 kg/m^2^ | +3 | 8 | 11 |
| Diagnosis or history of VTE | +8 | 5 | 6.8 |
| Thrombophilia^b^ | +8 | 0 | 0.0 |

^a^Other than surgery, respiratory surgery, urology, and gynecology

^b^Criteria included antiphospholipid antibody syndrome, hyperhomocysteinemia, increased prothrombin levels, factor V Leiden, increased factor VIII levels, hyperfibrinogenemia, antithrombin deficiency, protein C deficiency, and protein S deficiency

Abbreviations: CCDSS, computerized clinical decision support system; BMI, body mass index; VTE, venous thromboembolism
